# The scent of supercolonies: the discovery, synthesis and behavioural verification of ant colony recognition cues

**DOI:** 10.1186/1741-7007-7-71

**Published:** 2009-10-28

**Authors:** Miriam Brandt, Ellen van Wilgenburg, Robert Sulc, Kenneth J Shea, Neil D Tsutsui

**Affiliations:** 1 Department of Environmental Science, Policy and Management, 137 Mulford Hall #3114, University of California, Berkeley, CA 94720-3114, USA; 2 Project Management Jülich, Research Centre Jülich GmbH, 52425 Jülich, Germany; 3 Department of Zoology, University of Melbourne, Melbourne, Victoria 3010, Australia; 4 Department of Chemistry, 5042D Fredrick Reines Hall, University of California, Irvine, CA 92697, USA

## Abstract

**Background:**

Ants form highly social and cooperative colonies that compete, and often fight, against other such colonies, both intra- and interspecifically. Some invasive ants take sociality to an extreme, forming geographically massive 'supercolonies' across thousands of kilometres. The success of social insects generally, as well as invasive ants in particular, stems from the sophisticated mechanisms used to accurately and precisely distinguish colonymates from non-colonymates. Surprisingly, however, the specific chemicals used for this recognition are virtually undescribed.

**Results:**

Here, we report the discovery, chemical synthesis and behavioural testing of the colonymate recognition cues used by the widespread and invasive Argentine ant (*Linepithema humile*). By synthesizing pure versions of these chemicals in the laboratory and testing them in behavioural assays, we show that these compounds trigger aggression among normally amicable nestmates, but control hydrocarbons do not. Furthermore, behavioural testing across multiple different supercolonies reveals that the reaction to individual compounds varies from colony to colony -- the expected reaction to true colony recognition labels. Our results also show that both quantitative and qualitative changes to cuticular hydrocarbon profiles can trigger aggression among nestmates. These data point the way for the development of new environmentally-friendly control strategies based on the species-specific manipulation of aggressive behaviour.

**Conclusion:**

Overall, our findings reveal the identity of specific chemicals used for colonymate recognition by the invasive Argentine ants. Although the particular chemicals used by other ants may differ, the patterns reported here are likely to be true for ants generally. As almost all invasive ants display widespread unicoloniality in their introduced ranges, our findings are particularly relevant for our understanding of the biology of these damaging invaders.

## Background

Chemical signalling is the most ancient mode of communication and it is still used in some form by all extant organisms [1]. Insects, in particular, excel at the production and perception of chemical cues and rank as the best-studied model organisms in chemical ecology [2]. However, despite the ubiquity and importance of chemical communication, there are still many gaps in our knowledge of the specific chemicals involved. The elaborate social systems of ants, for example, are largely regulated by chemical signals, but very little is known about the chemical labels that define colony membership.

Many insects communicate using a class of chemicals known as cuticular hydrocarbons (CHCs). Although these waxy chemicals probably evolved as barriers to desiccation and microbial infection [3,4], they have since gained functions as cues for various types of recognition and communication (reviewed in [2,5,6]), including courtship [7], speciation [8] and parasitism via aggressive chemical mimicry [9].

The chemical cues that regulate membership in insect societies are also believed be cuticular hydrocarbons. In ants, for example, correlative analyses have shown that different colonies have distinctive differences in CHC profiles [10-12], and some specific CHCs have been implicated as candidate recognition cues, based on their association with colony boundaries and colonymate acceptance and rejection behaviours [10-14]. It is important to note, however, that although correlative approaches have highlighted promising candidate molecules as colony recognition cues, proving that specific compounds are responsible for colonymate discrimination has been very difficult. Candidate chemicals for colonymate recognition often occur in small amounts on tiny insects and the biochemical separation of individual compounds can be challenging or, in some cases, impossible. Moreover, the overall CHC profile can be a complex mixture of tens to hundreds of straight chain alkanes, alkenes and methyl-branched alkanes [15], and only a small subset of them are likely used as recognition cues. One recent study has suggested a particular dimethyl alkane as one of the colonymate recognition cues in the ant, *Camponotus herculeanus *[16]. Beyond this, however, little is known about which particular CHCs have been co-opted for colonymate recognition, or how individual social insects discriminate between the cocktails of odours that characterize members of their own colony and those of foreign colonies.

For Argentine ants, the identification of recognition cues is particularly important because altered social behaviour plays an important role in the ecological success of introduced populations. Argentine ants in South America, their native habitat, form colonies that range from a few meters in diameter up to several hundred metres long [17,18]. These colonies harbour high levels of genetic diversity, are genetically differentiated from each other and exhibit a pattern of genetic isolation by distance [19]. In contrast, introduced populations are genetically depauperate, genetically homogeneous and form geographically vast supercolonies that can extend for thousands of kilometres [20]. Experiments both in the laboratory and in the field have shown that this widespread cooperation in introduced populations plays a major role in their ability to displace the native species [21,22] which, in turn, can lead to negative indirect effects on other native taxa [23].

In this study, we first identified candidate hydrocarbons for colonymate recognition in the Argentine ant using several lines of evidence. We then developed and performed new methods for synthesizing seven of these molecules in a pure form. Next, we experimentally tested these chemicals for their ability to induce aggression among worker ants from the same colony. Finally, we tested the behavioural responses to changes in the quantity of these colonymate recognition cues and how combinations of different synthetic recognition cues affected the expression of aggression toward treated colonymates.

## Results and discussion

**Figure 1:**
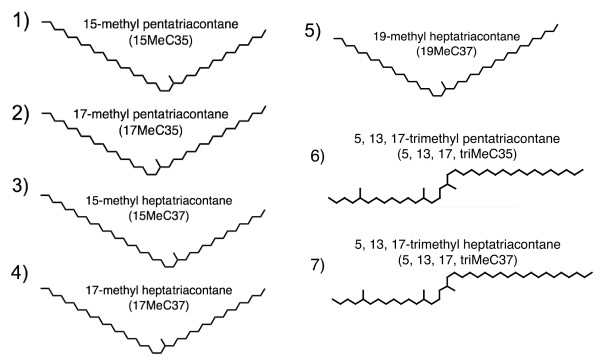
**Structures of the seven synthetic hydrocarbons identified as candidate recognition cues, synthesized and used in bioassays**.

**Figure 2:**
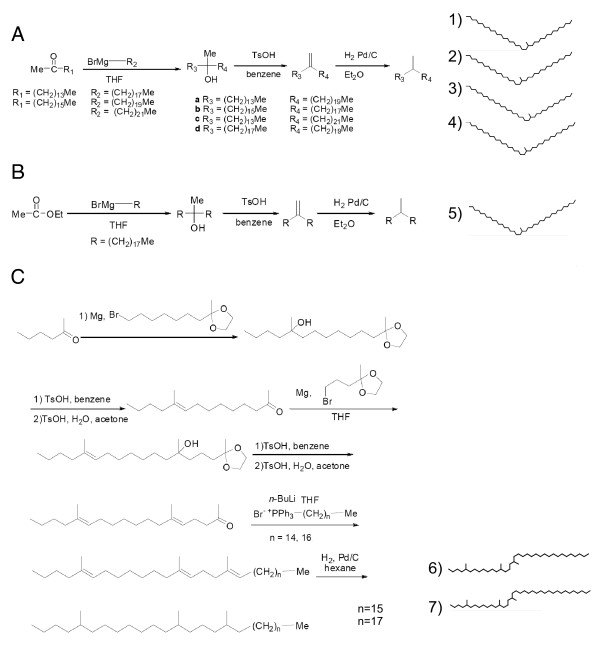
**Synthesis procedures for candidate recognition cues**. (A) Method of synthesis for the four asymmetric single-methyl alkanes (15MeC35, 17MeC35, 15MeC37, 17MeC37). (B) Method of synthesis for the symmetric single-methyl alkane (19MeC37). (C) Retrosynthetic analysis for the two tri-methyl alkanes (5, 13, 17triMeC35; 5, 13, 17triMeC37).

We first identified candidate recognition cues as the long-chain CHCs that differed in abundance between agonistic supercolonies. We chose five CHCs that differed between colonies for synthesis and behavioural testing and also selected two structurally similar CHCs (15-methyl pentatriacontane [15MeC35] and 17-methyl pentatriacontane [17MeC35]) for testing based on ease of synthesis (Figure 1). To test these chemicals as colonymate recognition cues, we developed procedures to synthesize these CHCs using a sequence of linear alkyl Grignard additions to methyl ketones followed by dehydration and hydrogenation steps to produce the saturated alkanes (Figure 2). We then mixed the synthetic hydrocarbons with CHCs extracted from colonymates, applied this mixture to living workers and quantified the behaviour of colonymates toward the treated workers [24]. We also performed two negative controls: the application of colonymate CHCs only and the application of colonymate CHCs spiked with an alkane (C36) which is not present in the Argentine ant profiles.

**Figure 3:**
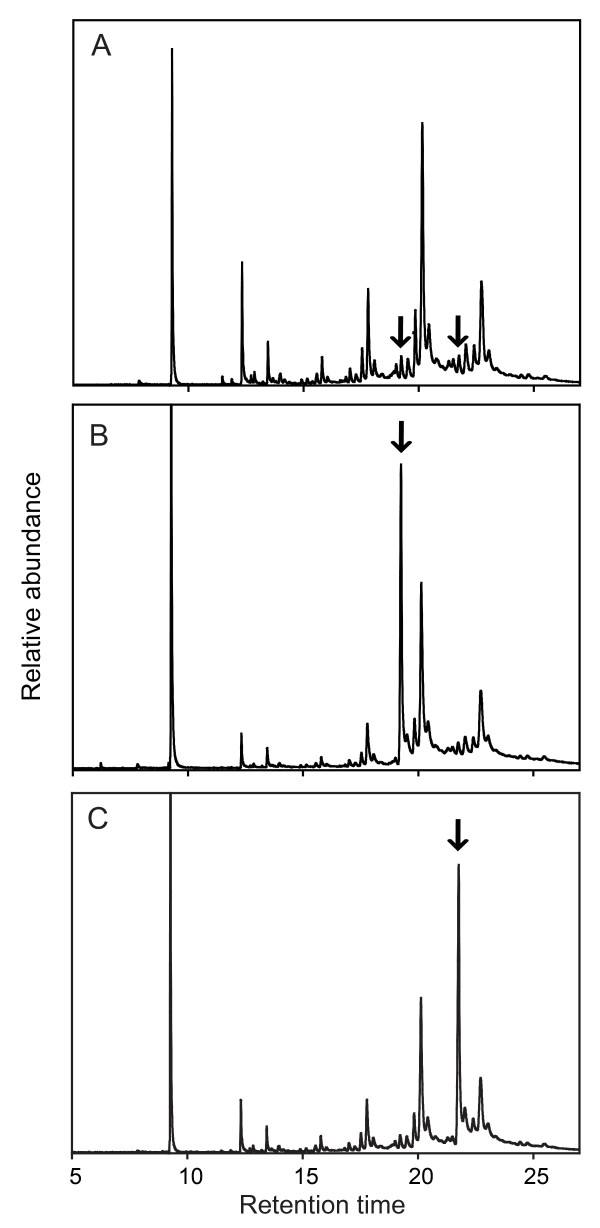
**Analysis of cuticular hydrocarbon (CHC) profiles using gas chromatography-mass spectrometry**. (A) Example chromatogram from control (colonymate CHC-treated) ants. (B) Example chromatogram from ants treated with a combination of the two synthetic single-methyl C35s (arrow). (C) Example chromatogram from ants treated with a combination of the three synthetic single-methyl C37s (arrow). Because a mixture of the synthetic CHCs was applied, the effective concentration of each individual CHC is a half and one-third of the peak size shown here, respectively.

We verified the presence and concentration of the synthetic hydrocarbons on the cuticle of treated ants by re-extracting CHCs from a subset of the treated ants and analysing their profiles using gas chromatography-mass spectrometry (GC-MS; Figure 3). As expected, the hydrocarbon profiles of the treated ants revealed that only the targeted hydrocarbon peaks were augmented by the treatment. Profiles from control ants (treated with colonymate CHCs; Figure 3A) did not differ from the profiles of unmanipulated ants.

**Figure 4:**
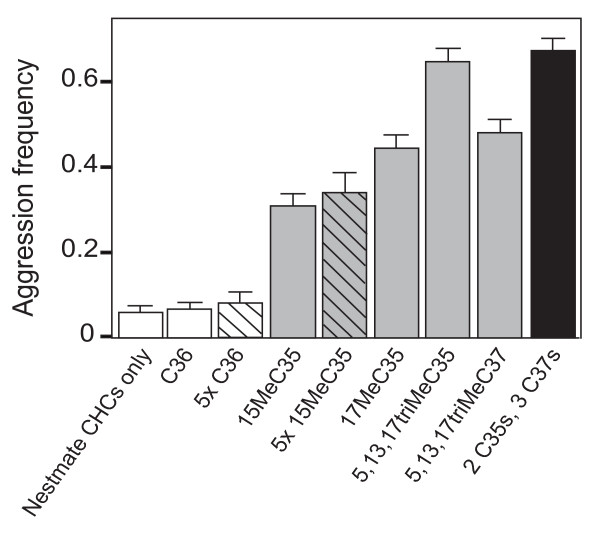
**Mean levels of aggression (± standard error) across all colonies for synthetic hydrocarbon treatments (grey and black bars) and controls (open bars)**. Aggression toward nestmates treated with five times the standard concentration (5×) of the control hydrocarbon (C36) and one of the synthetic hydrocarbons (15MeC35) are shown by the hatched open bar and hatched grey bar, respectively. Aggression toward nestmate ants treated with a combination of five different synthetic hydrocarbons is shown by the black bar.

**Figure 5:**
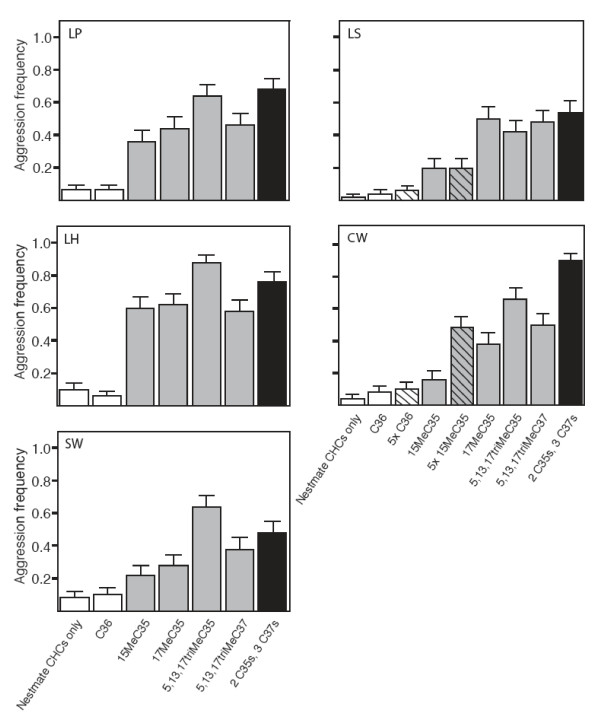
**Mean levels of aggression (± standard error) displayed by ants from each individual colony toward nestmates treated with synthetic hydrocarbons (grey and black bars) or controls (open bars)**. Color scheme follows that described for Figure 3. LP = Los Peñasquitos, LS = Lake Skinner, LH = Lake Hodges, CW = Cottonwood, SW = Sweetwater Reservoir.

Workers treated with synthetic hydrocarbons were attacked by their colonymates more frequently than were the control workers (Figure 4; overall *F*_8,1937 _= 40.05, *P *< 0.0001; Dunnett-Hsu, *P *< 0.001 for each individual comparison; *P *= 0.99 for C36 control). Importantly, the treatments elicited varied responses from different supercolonies (χ^2^, *P *< 0.01, Figure 5). This variation suggests that our synthetic hydrocarbons truly altered colonymate recognition rather than, for example, causing the treated ants to smell like insect prey (which should produce a uniformly aggressive reaction from all colonies). Instead, the variation in response across colonies suggests that each uses a different subset of hydrocarbons as recognition labels.

To test whether larger amounts of the hydrocarbons trigger higher levels of aggression, we tested the response to a fivefold increase in the concentration of 15MeC35, using ants from two of the supercolonies (Lake Skinner [LS] and Cottonwood [CW]). Overall, workers treated with this amount were attacked more frequently than ants treated with the standard concentration (Figure 4; *F*_1,197 _= 6.41, *P *< 0.05), suggesting that the amount of a particular recognition cue can be important for colony-mate recognition. However, when we analysed the results separately for each colony, we found that one of the colonies (CW) showed a significantly higher level of aggression toward colonymates treated with the fivefold concentration of 15MeC35 than the standard concentration, (χ^2^, *P *< 0.001, Figure 5), while the other colony (LS) did not (χ^2^, *P *= 1, Figure 5). These results indicate that the effect of CHC concentration on inter-colony aggression most likely depends on the initial CHC concentration in the profile of the colonymate. Unfortunately, because 15MeC35 co-elutes with other single methyl C35s we are unable to quantify the normally occurring concentrations of this CHC in the different colonies. Increasing the amount of C36 (the control hydrocarbon) fivefold did not trigger aggression (Figures 4, 5; overall Dunnett-Hsu *P *= 0.95). High concentrations of colonymate CHC extracts also do not trigger intracolony aggression [24].

Colonymates were attacked at even higher rates when a mixture of five different synthetic hydrocarbons were simultaneously applied, each at the same concentration as when applied individually, resulting in a fivefold increase in the total amount applied (Figure 4, black; *F*_1,197 _= 29.30, *P *< 0.001). When we analysed the results separately for each colony, aggression occurred more frequently towards colonymates with five different synthetic hydrocarbons than control colonymates (all colonies χ^2^, *P *< 0.001, Figure 5). Thus, both qualitative and quantitative changes to the hydrocarbon profile can trigger aggression among colonymates.

Our findings advance the understanding of these colonymate recognition systems by providing the first identification, chemical synthesis and behavioural testing of the cues used for Argentine ant colonymate recognition. Not only does the application of individual synthetic hydrocarbons trigger frequent aggressive rejection of colonymates, but our studies also demonstrate the importance of both qualitative and quantitative changes in CHC profiles.

In addition to the compounds examined here, it is likely that other CHCs are also used as recognition cues. Indeed, in our analysis of the natural hydrocarbon variation and aggressive behaviour, other individual chemicals appeared to vary among aggressive colonies, but not among geographically distant sites within the same supercolony. These CHCs merit further study.

The approach used here could be applied to future studies that seek to clarify the important functional features of these molecules. Chemical synthesis and careful testing of related hydrocarbons may provide insights into the mechanisms used to perceive them. For example, synthesis and testing of hydrocarbons that vary only in methyl group number, methyl group location or chain length may reveal which of these features are recognized by various receptor proteins of the chemosensory system. Similarly, future studies that couple electrophysiological techniques with the types of approaches used here may reveal how the detection and perception of chemical compounds differs from the behavioural response.

Our findings suggest potential control strategies for invasive ants based on colony-specific behavioural modification through the use of synthetic recognition cues. Since the striking ecological dominance of introduced populations stems from the widespread lack of aggression, synthetic colonymate recognition cues (such as those described here) could be used to initiate aggression among nestmates. As this approach would specifically target the social behaviour of only targeted ant species, such control strategies could reduce the harm to non-target organisms caused by the use of general insecticides. However, significant challenges regarding the method and efficacy of CHC delivery and potential long-term behavioural responses must first be addressed.

## Conclusion

The formation of cooperative colonies underlies the success of social insects generally, and of ants in particular. Although it has been well-established that ants can use CHCs as the cues for colony membership, the identity of the specific chemicals used has proven elusive. This study provides some of the first insights into the identity of ant colonymate recognition cues and illustrates how both qualitative and quantitative variation in these cues can affect social behaviours. Future studies will be able to apply these findings to the exploration of social evolution, sensory ecology, neurophysiology and invasion biology.

## Methods

### Identification of putative recognition cues

To identify the cuticular hydrocarbons that are the likely recognition cues, we combined multiple lines of evidence. Initially, we decided to seek recognition cues among the Argentine ant hydrocarbons that were 35 or 37 carbons long, based on the observation that CHCs of this size from the brown-banded cockroach (*Supella longipalpa*) appear to trigger intraspecific aggression among Argentine ants [25]. Next, we examined a previously unpublished dataset of the cuticular hydrocarbons from two particular populations: Davis, California (which is behaviourally part of the large Californian supercolony) and LS, California (which is a small, localized secondary colony) (Additional file 1). For our initial proof-of-concept experiments, we wanted to test single-methyl alkanes, due to their ease of synthesis. Based on the abundance and degree of between-colony variation, we chose 15-methyl heptatriacontane (15MeC37) and 17-methyl heptatriacontane (17MeC37) as the first candidates to test. We also chose to test 15MeC35 and 17MeC35 because they could be synthesized using the same reactions and starting materials, although these alkanes were naturally less abundant on the ants and less variable between colonies. After testing these compounds and discovering their ability to trigger intraspecific aggression, we decided to select a trimethyl alkane for synthesis and testing. To do this, we again examined the hydrocarbon profiles above, and reexamined previously collected data from a large analysis of cuticular hydrocarbon variation in natural populations of Argentine ants in California [11]. Based on these data, we chose to synthesize and test 5,13,17-trimethyl pentatriacontane (5, 13, 17triMeC35) and 5,13,17-trimethyl heptatriacontane (5, 13, 17triMeC37). It is likely that other CHCs not tested here can also function as recognition cues.

### Chemical synthesis of candidate recognition cues

The single methyl-substituted hydrocarbons (15MeC35, 17MeC35, 15MeC37, 17MeC37 and 19MeC37) were prepared by the addition of Grignard reagents derived from long chain alkyl halides to methyl ketones of the appropriate chain length (Figure 2). The resulting alcohols were dehydrated and subsequently hydrogenated to yield the methyl substituted saturated alkanes. The trimethyl derivatives (5,13,17triMeC35, 5,13,17triMeC37) were assembled by a multistep reaction sequence that involved the addition of a Grignard reagent incorporating a protected methyl ketone to a second unprotected methyl ketone (Figure 2). Deprotection and repetition of the sequence, followed by dehydration and hydrogenation, completed the syntheses. Intermediate and final reaction products were evaluated using GC-MS, ^1^H-NMR (nuclear magnetic resonance) and/or ^13^C-NMR. In all cases, mixtures of stereoisomers were produced and were used as such in the experiments.

### Behavioural testing of synthetic recognition cues

We performed behavioural assays using Argentine ants from sites belonging to five supercolonies in California: Los Peñasquitos (LP), LS, Lake Hodges (LH), CW and Sweetwater (SW). These supercolonies are behaviourally and genetically distinct from each other [17,26]. Ants at LP belong to the large supercolony that dominates the introduced range in California, whereas the other sites belong to much smaller secondary supercolonies. Ant colonies were maintained in the laboratory in tubs lined with Fluon and were fed a diet of sugar water, protein solution and scrambled eggs three times per week.

To determine the amount of synthetic hydrocarbon to apply, we constructed a calibration curve of hydrocarbon solution concentration versus peak area in the chromatograms, using eicosane (C20) as an internal standard. We re-extracted CHCs from treated ants in order to empirically determine the amount of synthetic hydrocarbon necessary for a two- to sevenfold increase (an amount that is within the range of naturally occurring CHC variation; see Figure 3).

We performed a total of 1950 individual behavioural assays (Figures 4, 5). All treatments were performed using 50 different workers from each supercolony, except the treatment with fivefold 15MeC35 and the fivefold C36 control, which were only performed with ants from LS and CW.

To apply the synthetic hydrocarbons, we extracted cuticular lipids from 200 workers, as in Torres *et al*. [24], and then added the designated amount of synthetic hydrocarbon solution (1 mg/ml hexane) to the CHC extract. The solvent was evaporated under nitrogen while swirling the vial, thus coating the walls of the vial with CHCs. We treated live ants by placing them in the vial, vortexing them, then allowing them to recover as in Torres *et al*. [24] and Liang and Silverman [27].

To quantify the behavioural response of colonymates, treated workers were tested against 10 members of their own colony in 35 mm Fluon-coated Petri dishes for 5 minutes. We scored behavioural assays as either aggressive (flaring of mandibles, recoil behaviour, biting, grabbing, use of chemical defenses) or non-aggressive.

In order to visualize the hydrocarbon profiles, we extracted CHCs from 10 treated workers, purified them on a silica column, and analysed them using GC-MS as in Torres *et al *[24].

In order to test the behavioural response to the hydrocarbon treatment, we used a mixed-model logistic-regression approach (SAS 9.1, Glimmix). We used the presence or absence of aggression as the response variable, the treatment as a fixed factor and the colony as a random factor. We performed several pre-planned comparisons between treatments using a Dunnett's test from the MIXED procedure of SAS. We selected a Dunnett's test because it allows the comparison of treatments with a reference. To test whether workers responded aggressively toward treated colonymates, we compared the frequency of aggression in each treatment to the negative controls. To test for the effect of the random term (colony) we ran the model twice, once with and once without the random term and compared the -2 Res Log Likelihood values in a χ^2 ^test.

## Abbreviations

C36: hexatriacontane; CHC: cuticular hydrocarbon; CW: Cottonwood; GC-MS: gas chromatography-mass spectrometry; LH: Lake Hodges; LP: Los Peñasquitos; LS: Lake Skinner; SW: Sweetwater;15MeC35: 15-methyl pentatriacontane; 17MeC35: 17-methyl pentatriacontane; 15MeC37: 15-methyl heptatriacontane; 17MeC37: 17-methyl heptatriacontane; 19MeC37: 19-methyl heptatriacontane; 5, 13, 17triMeC35: 5, 13, 17-trimethyl pentatriacontane; 5, 13, 17triMeC37: 5, 13, 17-trimethyl heptatriacontane.

## Authors' contributions

MB, KJS and NDT conceived and designed this study. RS and KJS devised and performed the chemical syntheses. MB and EvW conducted field-work, performed the behavioural assays and contributed equally to this paper. MB and EvW analysed the data. KJS and NDT provided funding for the research. EvW and NDT wrote the manuscript with input from all authors.

## Supplementary Material

Additional file 1 **Table S1 - Comparison of hydrocarbon profiles of Argentine ants from two different supercolonies, sample in Davis, California and Lake Skinner, California**. The value for each CHC is the percent area under the curve. The CHCs selected for synthesis and behavioral testing are boxed. Click here for file
