# Supplementary material for: The scent of supercolonies: the discovery, synthesis and behavioural verification of ant colony recognition cues
Source: BMC Biol. 2009 Oct 28;7:71. doi: 10.1186/1741-7007-7-71 (PMC2775022; doi:10.1186/1741-7007-7-71)
Supplement: Additional file 1 — Table S1 - Comparison of hydrocarbon profiles of Argentine ants from two different supercolonies, sample in Davis, California and Lake Skinner, California. The value for each CHC is the percent area under the curve. The CHCs selected for synthesis and behavioral testing are boxed. [file 1741-7007-7-71-S1.PDF]

Supplementary Table 1. Comparison of hydrocarbon profiles of Argentine ants from two different supercolonies, sample in Davis, California and Lake Skinner, California. The value for each CHC is the percent area under the curve. The CHCs selected for synthesis and behavioral testing are boxed.

Peak #	Hydrocarbon	Davis	Lake Skinner	Ratio
1	C19	0.12	0.19	0.65
2	C21	0.07	0.06	1.08
3	C23	0.22	0.31	0.69
4	C25	0.14	0.27	0.52
4.1	C26	0.03	0.07	0.48
5	C27	0.88	1.63	0.54
6	3meC27	0.09	0.15	0.60
7	C28	0.08	0.20	0.39
8	C29	0.64	1.12	0.58
9	15; 13; 11-meC29	0.02	0.13	0.13
10	7-meC29	tr	0.08	
11	5-meC29	tr	tr	
12	3meC29	0.05	0.11	0.45
13	5,11; 5,15-dimeC29	0.03	0.07	0.47
14	7,11,15-trimeC29		1.07	0.00
15	5,11,15-trimeC29		0.16	0.00
16	3,11,15-trimeC29		0.20	0.00
17	C31	0.20	0.84	0.24
18	15; 13-meC31	0.03	0.63	0.04
19	13,17; 11,15-dimeC31	tr	0.64	
	9,13-dimeC31		tr	
20	3-meC31	0.05	0.25	0.20
	7,11-dimeC31		tr	
21	5,13; 5,15; 5,17-dimeC31	0.10	0.34	0.29
22	9,13,17-trimeC31		0.86	0.00
23	7,11,15; 7,11,17-trimeC31		1.81	0.00
24	5,13,17-dimeC31	0.40	2.71	0.15
25	3,11,15; 3,11,17-trimeC31 + 12-meC32	0.04	1.07	0.04
26	14,18-dimeC32		0.37	0.00
27	4,16; 4,18-dimeC32	0.05	0.29	0.17
28	C33	0.21	1.03	0.21
29	4,14,18-trimeC32	0.07	0.21	0.31
30	17; 15; 13-meC33	0.36	2.61	0.14
31	15,19-dimeC33	0.25	1.30	0.19
32	9,13-dimeC33		1.09	0.00
33	5,15; 5,17-dimeC33	1.18	2.26	0.52
34	9,13,17-trimeC33		1.57	0.00
35	7,11,15; 7,11,17; 7,13,x-trimeC33		1.80	0.00
36	5,13,17; 5,15,19-trimeC33	3.70	7.36	0.50
37	3,11,15; 3,11,17; 3,13,17-trimeC33 + 14-meC34	0.60	2.21	0.27
38	14,18; 15,19-dimeC34	0.12	0.64	0.19
39	4,14; 4,16; 4,18; 4,20-dimeC34	0.20	0.46	0.43
40	C35 + 6,14,18-trimeC34	0.55	1.19	0.46
41	4,14,18-trimeC34	0.62	0.49	1.26
42	17; 15; 13-meC35	1.66	3.10	0.54
43	15,19; 13,17; 11,15-dimeC35	1.83	3.97	0.46
	9,13-dimeC35		tr	
44	5,15; 5,17-dimeC35	5.70	5.90	0.97

Peak #	Hydrocarbon	Davis	Lake Skinner	Ratio
	9,13,17-trimeC35		tr	
45	7,11,15-trimeC35		1.54	0.00
46	5,13,17; 5,13,19; 5,15,19-trimeC35	25.12	15.94	1.58
47	3,11,17; 3,11,19; 3,13,x-trimeC35	3.48	3.54	0.98
48	14,18-dimeC36	1.20	1.05	1.15
49	6,16-dimeC36	0.37		
50	4,14; 4,16-dimeC36	0.39		
51	6,14,18-trimeC36	1.69	1.15	1.46
52	4,14,18-trimeC36	1.49	0.53	2.79
	3,x,x-trimeC36		tr	
53	17; 15; 13-meC37	3.21	2.37	1.35
54	15,19; 13,17; 11,15-dimeC37	5.30	4.67	1.13
55	5,15-dimeC37	5.71	2.19	2.60
56	7,11,15-trimeC37		1.72	0.00
57	5,13,17; 5,15,x-trimeC37	20.37	8.02	2.54
58	3,11,17; 3,13,x; 3,15,x-trimeC37	4.02	2.07	1.94
59	14,18; 15,19-dimeC38	1.15	0.48	2.39
60	17; 15; 13-meC39	1.39	0.47	2.94
61	15,19; 13,17-dimeC39	1.78	0.80	2.24
62	5,17-dimeC39	0.57		
63	5,13,17; 5,15,19-trimeC39	1.87	0.61	3.07
64	3,x,x-trimeC39	0.58		
